# PPAR-*γ*, Microglial Cells, and Ocular Inflammation: New Venues for Potential Therapeutic Approaches

**DOI:** 10.1155/2008/295784

**Published:** 2008-03-11

**Authors:** Fiorella Malchiodi-Albedi, Andrea Matteucci, Antonietta Bernardo, Luisa Minghetti

**Affiliations:** ^1^Departmento di Ambiente e Connessa Prevenzione Primaria, Istituto Superiore di Sanità, 00161 Rome, Italy; ^2^G.B. Bietti Foundation for Ophthalmology (IRCCS), 00198 Rome, Italy

## Abstract

The last decade has witnessed an increasing interest for the role played by the peroxisome proliferator-activated receptor-*γ* (PPAR-*γ*) in controlling inflammation in peripheral organs as well as in the brain. Activation of PPAR-*γ* has been shown to control the response of microglial cells, the main macrophage population found in brain parenchyma, and limit the inflammation. The anti-inflammatory capacity of PPAR-*γ* agonists has led to the hypothesis that PPAR-*γ* might be targeted to modulate degenerative brain diseases in which inflammation has been increasingly recognized as a significant component. Recent experimental evidence suggests that PPAR-*γ* agonists could be exploited to treat ocular diseases such as diabetic retinopathy, age-related macular degeneration, autoimmune uveitis, and optic neuritis where inflammation has relevant role. Additional PPAR-*γ* agonist beneficial effects could involve amelioration of retinal microcirculation and inhibition of neovascularization. However, PPAR-*γ* activation could, in some instances, aggravate the ocular pathology, for example, by increasing the synthesis of vascular endothelial growth factor, a proangiogenic factor that could trigger a vicious circle and further deteriorate retinal perfusion. The development of new in vivo and in vitro models to study ocular inflammation and how to modulate for the eye benefit will be instrumental for the search of effective therapies.

## 1. INTRODUCTION

The peroxisome proliferator-activated receptor-*γ* (PPAR-*γ*) is a ligand-inducible transcription factor that belongs to a large superfamily comprising the nuclear receptors for steroids, thyroid hormones, and retinoids. The PPAR-*γ* and the two closely related PPAR-*α* and PPAR-*δ* (also known as *β*, NUC-1, or FAAR) are activated by naturally occurring fatty acids and act as sensors that regulate whole body metabolism in response to the dietary intake by controlling lipid and carbohydrate metabolism and lipid storage [1]. All three PPARs, once agonist-activated, form heterodimers with retinoic X receptors and regulate specific target gene transcription by binding to specific DNA regions (peroxisome proliferator response elements, PPREs) or by a mechanism independent of PPRE binding, termed transrepression, which begins to be unravelled [2].

Because of their role in the regulation of genes involved in lipid and carbohydrate metabolism, PPARs deeply affect lipid homeostasis and insulin sensitivity [3, 4]. The serum glucose lowering activity of PPAR-*γ* has lead to the development of specific PPAR-*γ* agonists for the treatment of type-2 diabetes and the metabolic syndrome [5]. PPAR-*γ* agonists such as thiazolidinediones (TZD), including pioglitazone (Actos) and rosiglitazone (Avandia), increase insulin sensitivity thereby improving glycaemic control, but also modify lipidemic profile and decrease blood pressure [6–9]. On the other hand, fibrates, which are PPAR-*α* agonists, are prevalently antilipidemic drugs, and therapeutic benefits of PPAR-*α* and PPAR-*γ* activations, which only are minimally overlapping, have generated interest in dual agonists that target both receptors, thus offering improved insulin sensitivity and lipidemic control in the same molecule [10, 11]. This would provide a therapeutic tool against diabetes and the metabolic syndrome.

The three PPARs share a high homology, but differ for tissue distribution and ligand specificity. PPAR-*α* is mainly expressed in tissues with high catabolic rates of fatty acids, such as the liver, muscle, and heart, whereas PPAR-*δ* shows a much wider distribution. PPAR-*γ* is highly expressed in adipose tissue, where it plays a central role in the regulation of adipocyte differentiation [12], and in cells of the immune system, including lymphocytes and macrophages. In peripheral monocytes, PPAR-*γ* expression is induced during the process of extravasation from blood vessels into the tissues, and in the course of activation by pro-inflammatory stimuli, suggesting that PPAR-*γ* is important for promoting monocyte-macrophage differentiation and activation and, thus, controlling inflammation [13–16]. As for macrophages of peripheral tissues, PPAR-*γ* regulates the activation of microglial cells, the main macrophage population found in brain parenchyma, and increasing evidence indicates that PPAR-*γ* might modulate brain inflammation and neurodegeneration [17] and be exploited as valuable therapeutic target in neurological diseases [18]. Indeed, brain inflammation is increasingly viewed as a target for treating neurological diseases, not only in classical infectious and immune-mediate disorders such as meningitis or multiple sclerosis, but also in stroke, trauma, and neurodegenerative diseases that were not originally considered to be inflammatory [19, 20].

In a similar way, inflammation could represent an important target to treat ocular diseases. In the study of ophthalmology, the classical subdivision of pathology textbooks in metabolic, inflammatory, hemodynamic, and degenerative disorders appears artificial and does not reflect the complexity of conditions, where inflammation, dysmetabolic and hemodynamic disorders, and neurodegeneration often conspire to the development of diseases. Paradigmatic example is diabetic retinopathy (DR), where a metabolic derangement (hyperglycemia) triggers a pathologic pathway, characterized initially by inflammation (leukostasis, enhanced retinal vascular permeability, Muller cell, and microglial activation), followed by microvasculature alterations and ischemia (proliferative DR), eventually leading to degeneration of neural retina and visual loss. To this complexity, a simplicity in the natural history may correspond and the course of different retinal diseases may at a certain stage converge toward a similar evolution. For example, pathologic neovascularization may be the same and ominous outcome of DR, age-related macular degeneration (AMD) and autoimmune uveitis, conditions that are very far from each other from the point of view of etiology.

In the present article, we will first briefly review the immune cells that participate to the ocular inflammation, mainly microglia, and the role of PPAR-*γ* in controlling their functions. In a second part, we will consider three conditions, where inflammation has a relevant function, microglia is involved, and the role of PPARs has been taken into consideration: DR, AMD, and optic neuritis (ON).

## 2. MICROGLIAL CELLS AND OTHER CELL POPULATIONS OF THE IMMUNE RESPOSE IN THE EYE.

Glial cells are the primary participants in the formation of scars in response to retinal or ocular injury and diseases. In addition, under normal conditions, they carry out a variety of supportive functions for the neurons with which they are closely related. Glial cells include astrocytes, oligodendrocytes, the retina-specific Muller-glial cells, and microglia, which are considered the main immune resident cells.

Retinal microglia, like their counterpart in the brain, belong to the myeloid lineage and their myeloid progenitors enter the nervous system primarily during embryonic and fetal periods of development. During embryogenesis, microglial precursors migrate to the retina before retinal vascularization and differentiate into ramified, quiescent microglia typical of adult healthy retina. A second population of phagocytes, which express macrophage markers, invades the retinal later through the developing vasculature and remains associated with the blood vessels (see below). In the adult retina, microglia are distributed through most of the retinal layers, including outer plexiform layer, outer nuclear layer, inner plexiform layer, ganglion cell layer, and nerve fiber layer. Engraftment experiments have shown that they display some proliferative capacity and have a slow turnover in respect of other macrophage populations [21]. Disturbances in the number or distribution of these cells disrupt the normal development of the eye and its related structures. Ritter and collaborators [22] have recently reported that myeloid progenitors migrate to vascular regions of the retina where they differentiate into microglia and facilitate the normalization of the vasculature, thus underlining a main role of microglial cells in promoting and maintaining retinal vasculature during development.

Microglia show particular capacity of interaction with retinal cells, supervising the immune environment (see [23] and references therein). As for microglia in the brain parenchyma, retinal microglial cells are immunocompetent cells, able to remove the debris created during normal eye development or degenerative conditions by phagocytosis and to mount an inflammatory and immune response against ocular injury, infection, and disease.

Under normal conditions, microglia are characterized by a downregulated phenotype when compared to other macrophage populations of peripheral tissues. The maintenance of microglia in this “inhibited” state is crucial for the regulation of the immune state of the retina, which has to maintain tissue homeostasis while preventing the destructive potential of inflammatory and immune response. The complexity of the several intraocular structures on which the correct vision is dependent renders the eye particularly vulnerable to the reactions of the immune system against invading pathogens or ocular injury. To prevent that a defensive reaction can transform into a threat to vision in itself, the eye is equipped with several regulatory mechanisms, which contribute to make the eye an “immune-privileged” site [24]. As recently described for the brain parenchyma [25], the immune privilege is not an absolute or an immutable state, but rather it is the result of the active interplay among specialized cellular elements and specific microenvironment characteristics, and it can be overcome in several instances. Among the main features that account for the ocular immune privilegeareisthe presence of blood-ocular barriers (the blood-aqueous barrier and the blood-retinal barrier), which are physical barriers between the local blood vessels and most parts of the eye itself, and the peculiar characteristics of the resident immune cells, namely, microglia, which are largely dependent on the presence of immunomodulatory factors in the aqueous humor and on the cross-talk between microglia and retinal cells. Several “ligand-receptor-” type interactions between retinal cells and microglia contribute to maintaining microglia in a nonactivated state. Among these, the glycoprotein CD200, which in the retina is extensively expressed in neurons and endothelial cells, and the cognate ligand CD200L on microglia [39], and the neuronal chemokine fractalkine (or CX3CL1) and its microglial receptor CX3CR1 [40].

In spite of their apparent “dormant” state, resting microglia actively monitor the surrounding microenvironment with extremely motile processes and protrusions, entering in contact with other cellular elements and sensing alterations in the nearby environment, to which they rapidly react. Microglial activation comprises morphological changes, such as cellular hypertrophy, retraction of processes, and expression of surface markers, as well as functional changes, including proliferation, migration, phagocytosis, and production of bioactive molecules. Activated microglia have been described in several forms of retinal injury or disease (see Table 1), in which they are believed to play major roles, either protective or detrimental. Indeed, activated microglia can, on one side, remove the degenerating neurons and contribute to re-establish tissue integrity; on the other side, they can secrete proinflammatory cytokines such interleukin (IL)-1*β*, IL-3, IL-6, tumour necrosis factor (TNF)-*α*, and interferon (IFN)-*γ*, which can be toxic to neurons and photoreceptors [41, 42] or to other cellular targets such as oligodendrocytes [43, 44]. In addition, several of these microglial products can up-regulate the expression of vascular cell adhesion molecules and chemokines [45–47], thus promoting the recruitment of lymphocytes and macrophages, and enhancing the immune-mediated tissue damage [23, 48]. In this context, molecules that can enable the control of microglial activation represent valuable tools to counteract the detrimental effects of inflammation and immune response while fostering those necessary for healing.

In addition to microglia, other cell types contribute to the immune response in the eye. The perivascular macrophages reside outside the blood-ocular barrier, in the space that separates the endothelium of the retinal capillaries and retinal pigment epithelium (RPE). Because of their anatomical location, they escape the tight control to which retinal microglia are subjected and their morphology and immunophenotype are very similar to those of macrophages of peripheral tissues. In close proximity, but separate from perivascular macrophages are the pericytes, which are believed to be essential as structural support in microcirculation. In addition, together with astrocytes and Muller glia, they are considered to play a major role in maintaining the inner blood-retinal barrier [49]. These cells, of mesodermal origin, are enclosed within the basal lamina on the abluminal surface of endothelial cells and contain contractile proteins. Pericytes have been shown to control vessel constriction and retinal blood flow [50], and are involved in several pathological conditions, including hypoxia, hypertension, and DR. Their activation, since the very early phases of disease, is thought contribute to the disruption of the blood-retinal barrier [51]. Finally, the RPE cells are important in ocular immune response and in maintaining the eye immune privilege. These cells form a monolayer between the neuroretina and the choroids and are the essential component of the outer blood-retinal barrier. One of the main characteristics of RPE cells is the presence of tight junctions at the apical side of their lateral membrane, which render the monolayer impermeable for macromolecules and limit access of blood components to the retina. In addition to several important supportive functions, including regulation of transport of nutrients to the photoreceptors, phagocytosis of damaged or old rod outer segments, and production of growth factors, RPE cells contribute to the immune and inflammatory response of the retina by expressing major histocompatibility complex (MHC) antigens, adhesion molecules, and a variety of cytokines, which may either promote or enhance immune responses or down-regulate them [52].

In addition to the cell types so far described, a novel population of dendritic cells has been recently reported in normal mouse retina, distinguishable by the cell types by the extent of specific surface antigens and anatomical tissue location [53].

## 3. DIABETIC RETINOPATHY

Diabetic retinopathy (DR) is one of the most serious complications of diabetes and the leading cause of blindness among working-age adults. DR symptoms are mostly due to the vascular alterations that affect the retina. The early events are increased blood flow and abnormal vessel permeability, due to the impairment of blood-retinal barrier. They are caused by hyperglycemia and the other metabolic consequences of excess glucose disposal. As the disease progresses, retinal vasculopathy develops, showing loss of pericytes, smooth muscle and endothelial cell death, and microaneurysm formation, resulting in areas of ischemia in the retina. At this stage, up-regulation of proangiogenic factors in ischemic retina, such as vascular endothelial growth factor (VEGF), initiates a vicious circle of neovascularization (proliferative DR), characterized by enhanced vascular leakage and formation of new, weak, and prone-to-break blood vessels, which further deteriorates retinal perfusion, worsens ischemia and eventually leads to visual loss.

Although the pathogenetic cascade connecting these events is still unclear, evidence suggesting a role for inflammation in DR is accumulating, supporting the involvement of both chemical mediators and inflammatory cells in the pathogenesis of the disease [54]. Elevated levels of proinflammatory cytokines, such as IL-1*β*, IL-6 and IL-8, and TNF-*α* and vascular cell adhesion molecule-1, have been found in the vitreous of patients with proliferative DR [55–57]. Increased VEGF and IL-6 levels were detected in the aqueous humor of diabetic patients with macular edema [58]. TNF-*α* was found in epiretinal membranes of proliferative DR [59]. Data from experimental models are in line with these observations. In streptozotocin (STZ)-induced diabetic rats, changes in retinal blood vessel permeability, which characterizes the early phases of DR, are paralleled by increase in the level of the intercellular cell adhesion molecule-1 (ICAM-1), which facilitates the trafficking of leukocytes [60], and pro-inflammatory mediators, such as TNF-*α* and cyclo-oxygenase-2 (COX-2) [61, 62]. In the same animal model, an increased level of IL-1*β* has been observed and put in relation to upregulated inducible nitric oxide synthase (iNOS) [63]. Mice deficient in the leukocyte adhesion molecules CD18 and ICAM-1 demonstrate significantly fewer adherent leukocytes in the retinal vasculature after induction of diabetes with STZ [54]. According to some authors, VEGF could be responsible for the initiation of the inflammatory cascade, as its administration in vivo was found to induce retinal ICAM-1 and endothelial NOS (eNOS) expression [64, 65]. As far as inflammatory cells are concerned, microglia seem to be mostly involved. Microglial activation appears early in the course of DR, before the onset of overt neuronal cell death [62]. In STZ-induced diabetic rats, hypertrophic microglia were observed one month after the onset of diabetes [66], with significant increase also in cell number [67]. In mice with alloxan-induced diabetes, changes in microglial cell morphology were the first detectable cellular modifications, apparently preceding ganglion cell apoptosis and increase in blood barrier permeability [68]. Treatment of STZ-induced diabetic rats with minocycline, a semisynthetic tetracycline that counteracts microglial activation, besides decreasing the expression of proinflammatory cytokines, decreased caspase-3 levels [62], suggesting a potential neuroprotective antiapoptotic effect of inhibition of microglial activation.

Considering the role of inflammation in the pathogenesis of DR, it has been suggested that PPAR-*γ* ligands exert therapeutic effects also as modulators of inflammation, besides providing glycemic control [69]. In diabetic patients, PPAR-*γ* agonists reduce several markers of inflammation, such as serum levels of C-reactive protein, IL-6, monocyte chemoattractant protein-1 (MCP-1), plasminogen activator inhibitor-1, soluble CD40 ligand, and matrix metalloproteinase-9 [70–75]. In addition, they have been shown to induce the suppression of activated NF*κ*B and decrease ROS generation in blood mononuclear cells [70, 73].

Modulation of the inflammatory process has also been studied in DR in in vivo models. In streptozotocin-induced DR, rosiglitazone was shown to inhibit both retinal leukostasis and retinal leakage [76]. The effect was not accompanied by downregulation of proinflammatory cytokines, such as TNF-*α*, although the adhesion molecule ICAM was found reduced. Nitric Oxide (NO) of endothelial origin regulates ocular blood flow. In the endothelial dysfunction, which characterizes the early stages of DR, a reduction in the bioavailability of NO may contribute to impairment of ocular hemodynamics [77]. In bovine aortic endothelial cells, troglitazone increased NO production in a dose- and time-dependent manner with no modifications in eNOS expression [78]. A study focused on NO production in pericytes showed that PPAR-*γ* is constitutively expressed in retinal pericytes and that troglitazone increases NO production and iNOS expression in a PPAR-*γ*-dependent manner, an effect which is opposite to what observed in cultured microglia [79, 80]. This study suggests that PPAR-*γ* agonists, in addition to improving insulin sensitivity, might also improve retinal microcirculation in early DR [81]. However, NO is a double-edged sword. Overproduction of NO by neuronal NOS is supposed to contribute to retinal injury in ischemia [82, 83]. Thus, although in DR early phase an increase in NO may contribute to the improvement of retinal microcirculation, in proliferative DR a beneficial effect is doubtful. A further reason of concern is represented by TZD effects on VEGF. Several in vivo and in vitro studies have reported increased expression of VEGF in response to PPAR-*γ* ligands. TZDs have been found to upregulate VEGF in human vascular muscle cells [84], in 3T3-L1 adipocytes [85], in cultured cardiac myofibroblasts [86]. In bovine aortic endothelial cells treated with troglitazone, NO increase was accompanied by upregulation of VEGF and its receptor, KDR/Flk-1 [78]. Administration of pioglitazone [87] and troglitazone [85] also significantly increased plasma VEGF levels in diabetic patients. Considering the role played by VEGF in the development and progression of DR, caution has been suggested in the use of PPAR-*γ* ligands in patients with advanced disease [85, 87]. However, in partial disagreement with the results above reported, antiangiogenic properties of PPAR agonists have been shown both in in vitro and in vivo models [35, 88–90]. In neonatal mice, where ischemia was used as a model of retinal neovascularization, intravitreous injection of rosiglitazone or troglitazone inhibited development of new retinal vessels [91]. In the same study, TZDs have been found to inhibit retinal endothelial cell proliferation, migration, and tube formation in response to VEGF treatment [91]. Further studies are therefore required to clarify the issue.

## 4. AGE-RELATED MACULAR DEGENERATION

Age-related macular degeneration (AMD) is the leading cause of vision loss in the elderly in the western world. It is characterized by degeneration of the macula, the central area of the retina with the highest concentration of cone photoreceptors, responsible for visual acuity and color vision. Histopathologically, the early phase of AMD is characterized by formation of drusen, deposits of lipid and cellular debris that are found between the RPE cells and Bruch's membrane, possibly as a result of RPE degeneration or, as recently proposed [92], microglial infiltration and transformation in foam cells. As the disease proceeds, photoreceptor degeneration and, in the most aggressive cases, choroidal neovascularization (CNV) intervene, with growth of new blood vessels from the choroids into the subretinal space. Two major clinical phenotypes of AMD are recognized: nonexudative (dry type), and exudative (wet type). The latter more frequently develops into CNV.

AMD is a complex, multifactorial disease and both genetic and environmental factors may contribute at some level. In the pathogenesis of the disease, both altered angiogenesis and inflammation play a role. The study of pathologic angiogenesis in the retina has focused on two main factors: the angiogenic VEGF [93, 94] and the antiangiogenic PEDF [94–96], although a number of other factors are implicated(for a review, see [97]). It is widely agreed that in CNV an imbalance between angiogenic and anti-angiogenic factors takes place, but what disrupts this delicate equilibrium is still unclear. Several lines of evidence point to inflammation as a pathogenetic mechanism. Many risk factors for AMD are related to inflammation, including environmental factors, such as smoking and low intake of omega-3 fatty acid [98, 99], and genetic factors, such as polymorphisms of complement factor H [100–102] and the chemokine receptor CX3CR, which is expressed by microglia and mediates migration and adhesion in response to its ligand fractalkine or CX3CL1 [103]. Increased serum levels of IL-6 and C-reactive protein have been found to be related with progression of AMD [104]. More recently, IL-6 receptor neutralization has shown to lead to decrease in the expression of inflammatory mediators, such as the chemokine MCP-1, the adhesion molecule ICAM-1, and VEGF, and to reduce macrophage infiltration into CNV in in vivo model of the disease [105]. Inflammatory mediators, such as macrophage chemoattractants and activated complement components, especially C3a and C5a, are also found in drusen samples from AMD patients [106–108]. A role for complement in the development of the disease has been suggested [34]. In line with this hypothesis, it has been observed that genetic ablation of receptors for C3a or C5a reduced VEGF expression, leukocyte recruitment, and CNV [109].

Activation of microglia and infiltration of macrophages have been reported in the human AMD as well as in experimental CNV [110–112]. In transgenic mice lacking CX3CR1, microglia migrate defectively and accumulate in the subretinal space, evoking morphological and pathological features similar to those observed in human AMD. In addition, laser-induced CNV was exacerbated in these mice [92]. A controversy exists regarding the origin of activated retinal mononuclear phagocytes, that is, whether they are resident microglia [113, 114] or blood-derived bone marrow macrophages [46, 115]. In support of the latter hypothesis, it should be noted that systemic depletion of macrophages using clodronate-filled liposomes blocked neovascularization [116, 117]. However, the role of macrophages is still debated, since some studies suggest an antiangiogenic role for macrophages. For example, mice lacking CC chemokine ligand 2 (CCL2) or its receptor, both involved in chemoattraction of macrophages and/or microglia, show drusen-like deposits and CNV, suggesting that macrophage recruitment may protect against AMD [118]. In addition, mice lacking IL-10, an anti-inflammatory cytokine known to control macrophage/microglia functions, had significantly reduced neovascularization and increased macrophage infiltrates compared to wild type, in a laser-induced model of CNV. In these experiments, prevention of macrophage entry into the eye promoted neovascularization while direct injection of macrophages significantly inhibited CNV.

As mentioned earlier, beside mononuclear phagocytic cells, RPE cells have also a role in the inflammatory and angiogenetic process, as a major source of VEGF and PEDF. In addition, there is a cross-talk between RPE and macrophages. It has been shown that macrophages in CNV are immunopositive for VEGF, TNF-*α*, and IL-1*β* [119]. The latter factors can induce the secretion of IL-8 and MCP-1 in RPE cells in vitro [120, 121]. MCP-1 is, in turn, involved in the recruitment of macrophages [122], thus closing the circle. Indeed, in surgically excised CNV specimens, RPE was found to express VEGF and MCP-1 and macrophages were immunolabeled for VEGF [123].

The interest in the role of PPARs in AMD has been mainly focused on their activities as modulators of angiogenesis. PPAR agonists have shown antiangiogenic properties both in in vitro and in vivo models [35, 88, 89]. It has been shown that choroidal ECs and RPE cells express PPAR-*γ* and that PPAR-*γ* ligands inhibit their response to VEGF, without apparent toxicity to the adjacent retina, in a laser-induced model of CNV [90]. Decrease in angiogenesis apparently takes place by inhibition of VEGF, since PPAR-*α* agonists are found to inhibit endothelial VEGFR2 expression [124]. An opposite role has been recently described for PPAR*δ*, which induced endothelial proliferation and angiogenesis in vitro, through a VEGF-dependent mechanism [125]. The natural ligand 15-deoxy-Δ^12,14^-prostaglandin J_2_ (15d-PGJ_2_) was found to protect a human RPE cell line from oxidative stress by elevating GSH and enhancing MAPK activation, but such activity was independent of its PPAR-*γ* binding activity [126]. The roles of infiltrating macrophages and/or resident microglia in the pathogenesis of AMD open the possibility that PPAR-*γ* agonists may ameliorate the course of the disease also through the down-regulation of several proinflammatory functions of these cells [8] and reference therein, including TNF-*α* and iNOS, and MHC-II expression.

However, possible beneficial effects of PPAR-*γ* agonists in the treatment of ocular inflammation and, particularly, of AMD need to be further verified. It is important to keep in mind that PPAR-*γ* is involved in the differentiation of macrophages to foaming cells and PPAR-*γ* ligands can induce expression of adipocyte lipid binding protein (ALBP/aP2), a gene that is highly expressed in vivo in macrophage/foam cells of human atherosclerotic plaques [127]. Moreover, activation of PPAR-*γ* has been shown to reduce CCR2 expression in monocytes and their chemotaxis in response to MCP-1 [128]. These PPAR-*γ* mediated activities are of particular interest in the view of the recent finding by Combadière et al. [92], suggesting that subretinal microglial foam cells might be the origin of drusen-like deposits and that accumulation of microglia in the subretinal space may be a driving force in the pathogenesis of AMD.

## 5. OPTIC NEURITIS AND RELATED DISORDERS

Optic neuritis (ON), an inflammatory, demyelinating disease of the optic nerve, may be the initial symptom of multiple sclerosis (MS) or appear in the course of the disease. In any event, nearly half of MS patients develop ON during the course of the disease. An idiopathic demyelinating disorder of the optic nerve also occurs as NeuroMyelitis Optica (NMO) or Devic's disease, which is characterized by the coexistence of usually bilateral and severe optic neuritis with spinal cord involvement and the presence of a highly specific serum autoantibody marker (NMO-IgG), recognizing the transmembrane channel Aquaporin 4 [138, 139]. The boundaries between NMO and MS are, however, rather imprecise, from both the clinical and pathologic points of view and it is still a matter of controversy whether NMO should be considered a variant of MS or a separate entity [139, 140].

Considering their role in inflammation, the possible therapeutic efficacy of PPAR-*γ* agonists has been studied in experimental autoimmune encephalomyelitis (EAE), an animal model of the disease where the autoimmune reaction against myelin is induced in animals by active sensitization with myelin components. Although several criticisms have been moved towards this model, EAE still provides a valuable tool for improving our understanding on the pathogenesis and treatment of MS. EAE is also considered a model relevant to the study of demyelinated diseases of the optic nerve [141, 142]. An additional animal model is represented by T cell receptor transgenic mice specific for myelin oligodendrocyte glycoprotein (MOG). These mice develop isolated optic neuritis either spontaneously or after sensitization with suboptimal doses of MOG [143]. Therapeutic efficacy of PPAR-*γ* ligands has been demonstrated in terms of suppression or amelioration of clinical symptoms and decrease of inflammatory signs (see Table 2). Although the anti-inflammatory activities of PPAR-*γ* agonists are complex and multifaceted, evidence has been provided suggesting a direct action of PPAR-*γ* agonists on microglia/mononuclear phagocytic cells. Indeed, taking part in both innate and adaptive immune responses, microglia and mononuclear phagocytes are deeply implicated in the complex inflammatory cascade associated with MS. Their role has been recently and extensively reviewed [144, 145]. The PPAR-*γ* natural agonist 15d-PGJ_2_ [146] and the PPAR-*α* agonist gemfibrozil [133] were found to significantly reduce macrophage infiltration in the lesions. A decreased number of IL-1*β*-positive cells were found in EAE brain of mice treated with GW0742 and a PPAR-*δ* agonist and this observation was considered indicative of a reduction of glial activation [134]. PPAR-*γ* inhibition of microglial cell activation is also supported by in vitro experiments [8, 79, 80, 147–152].

Notwithstanding the amount of data regarding a therapeutic activity of PPAR agonists in EAE, clinical studies are lacking and report on their clinical use in MS or ON is still anecdotical [153]. Clinical trials are however in course with pioglitazone and rosiglitazone.

## 6. CONCLUSIONS

The promising results obtained in experimental models of ocular diseases and the recent advancements in the knowledge of the pathogenic mechanisms driving ocular damage and vision loss strongly point to PPAR-*γ* as a valuable target to control inflammation and treat invalidating diseases such as DR, AMD, and ON. Given the complexity of the phenomena that can be influenced by PPAR-*γ* activation, involving not only inflammation but also retinal microcirculation, neovascularization, and transformation of activated microglia in foam cells contributing to drusen-like deposits, further studies are mandatory for a correct evaluation of pro and cons of using PPAR-*γ* agonists in ocular disease treatment. The PPAR-*γ* agonists could also find other important applications in controlling the adverse effects of inflammation that can put at risk the eye integrity and the correct vision. As an example, some of the adverse reactions described after liquid artificial vitreous replacement use in vitreoretinal surgery are a consequence of inflammatory reaction and activation of mononuclear phagocytic cells [154], suggesting that the use of PPAR-*γ* agonists could be very advantageous in controlling the inflammatory response to biomaterials.

## Figures and Tables

**Table 1:** Retinal pathologies characterized by microglial activation.

Pathology	References
Diabetic retinopathy	[26–29]
Glaucomatous optic nerve degeneration	[30–33]
Human retinitis pigmentosa	[34]
Age-related macular degeneration	[34, 35]
Retinal ischemia and reperfusion injury	[36, 37]
Retinal degeneration	[14, 20, 38]

**Table 2:** PPAR agonists and EAE.

Agonists	Biological activity	Receptor	References
Troglitazone	Amelioration of clinical symptoms. Reduced expression of proinflammatory cytokines, IL1*β* and TNF-*α*	PPAR-*γ*	[129]
Ciglitazone, 15d-PGJ_2_	Decrease of severity and duration of clinical paralysis. Decrease of CNS inflammation and demyelination. Decrease of IL-12 production	PPAR-*γ*	[130]
15d-PGJ_2_	Delay in the onset and decrease in the severity of disease. Reduction of Con A- and MBP Ac1–11-reactive, IFN-*α*- and IL-4-secreting cells	PPAR-*γ*	[131]
Pioglitazone	Decreased mRNA levels of iNOS and the chemokines MIP1 and RANTES in the central nervous system	PPAR-*γ*	[132]
Gemfibrozil and fenofibrate	Dose-dependent suppression of lymphocyte proliferation. Promotion of IL-4 production and inhibition of IFN-*γ* production	PPAR- *α*	[133]
GW0742	Improvement of clinical recovery. Reduction of glial activation	PPAR- *δ*	[134]
Ciglitazone, 15d-PGJ_2_	Amelioration of clinical and pathological symptoms. Inhibition of neural antigen-specific T cell proliferation	PPAR-*γ*	[135]
Gemfibroil	Reduction of incidence and clinical signs. Inhibition of the infiltration of inflammatory cells into the CNS. Reduced expression of proinflammatory molecules such as iNOS, IL-1, IL-6, and TNF-*α*	no PPAR-*γ*	[136]
Pioglitazone	Prevention of relapse episodes and reduction of mean clinical scores during the treatment period. Decrease of IFN-*γ* levels	PPAR-*γ*	[137]
